# Twenty-Four-Hour Variation of Intraocular Pressure in Primary Open-Angle Glaucoma Treated with Triple Eye Drops

**DOI:** 10.1155/2017/4398494

**Published:** 2017-05-03

**Authors:** Yoshinori Itoh, Kenji Nakamoto, Hiroshi Horiguchi, Shumpei Ogawa, Takahiko Noro, Makoto Sato, Tadashi Nakano, Hiroshi Tsuneoka, Noriko Yasuda

**Affiliations:** ^1^Department of Ophthalmology, Jikei University School of Medicine, Tokyo, Japan; ^2^Department of Ophthalmology, Tokyo Metropolitan Police Hospital, Tokyo, Japan; ^3^Department of Ophthalmology, Nippon Medical School, Tokyo, Japan; ^4^Department of Ophthalmology, Atsugi City Hospital, Atsugi, Japan; ^5^Tokyo Metropolitan Institute of Medical Science, Tokyo, Japan; ^6^Department of Ophthalmology, Niizashiki Central General Hospital, Tokyo, Japan; ^7^Department of Ophthalmology, Showa University School of Medicine, Tokyo, Japan

## Abstract

*Objectives.* To evaluate 24-hour intraocular pressure (IOP) variation in patients with primary open-angle glaucoma (POAG) treated with triple eye drops. *Subjects and Methods.* The IOP was measured in 74 eyes in 74 POAG patients (seated) on triple therapy (PG analogue, *β*-blocker, carbonic anhydrase inhibitor) at about every 3 hours. *Results.* The peak IOP was 13.5 ± 3.1 at 1:00, and the trough IOP was at 12.6 ± 2.4 mmHg at 7:00. The IOP at 7:00 was significantly lower than that at 10:00, 1:00, and 3:00 (*p* < 0.05). Based on the time of the peak IOP, we classified the patients into two groups: diurnal (28 eyes) and nocturnal types (37 eyes). There was significant difference at the spherical equivalent between diurnal and nocturnal types (*p* = 0.014). To assess the influence of reflective error, we conducted subanalysis for two groups: high myopic (26 eyes, ≤−6D) and low/nonmyopic (24 eyes, ≥−2D) groups. In the low/nonmyopia group, the IOP was significantly higher at 1:00 and 3:00 than at 13:00, 16:00, and 7: 00 (*p* < 0.05). *Conclusion.* The mean of IOP elevated outside of clinic hour in the POAG patients on triple therapy. The low/nonmyopia patient should be carefully treated because the IOP of the patients at night elevated significantly.

## 1. Introduction

The intraocular pressure (IOP) reduction is the only evidence-based treatment for glaucoma [1–4]. The IOP in glaucoma patients is generally evaluated in single IOP measurement during clinic hours, although IOP varies over the course of 24 hours. Hence, it is obvious that understanding 24-hour IOP variation is important in glaucoma treatment.

Approximately 100 years ago, Maslenikow reported that IOP was generally higher during daytime than nighttime [5]. The biological clock in the suprachiasmatic nucleus, the central clock that regulates the circadian rhythm, controls aqueous humor production via sympathetic nervous system. Therefore, the IOP variation due to aqueous humor production is less at night than during daytime [6–9]. In general, the IOP variation pattern in the sitting position peaks in the morning and decreases toward midnight [10]. However, the IOP variation is affected by various factors such as posture and spherical equivalent [11].

In primary open-angle glaucoma (POAG), generally, the first-line treatment to reduce IOP is an instillation of eye drops. Daytime peak IOP is clinically important in predicting long-term glaucomatous progress in the patients treated with one or two kinds of eye drops because the IOP peaks at night in only 20% of cases [12]. In some cases, however, visual field defects progress quickly in spite of adequate reduction of IOP measured during clinic hours. The next step of treatment is usually to add eye drops up to three or four different types. Importantly, combinations of eye drop treatment affect the pattern of the IOP variation. In many cases, the IOP during eye drop treatment peaks outside clinic hours [13, 14]. Moreover, the effective IOP-lowering durations vary among eye drops, which may also lead to the IOP variation. Unlike *β*-blockers, prostaglandin analogs (PG) and carbonic anhydrase inhibitor (CAI) lower the IOP significantly throughout 24 hours. During *β*-blocker treatment, the IOP is higher during daytime than at night [15–17].

When treated with multiple eye drops, combinations of drops generally flatten the IOP variation [18, 19]. Nakakura et al. found that the 24-hour IOP in patients treated with multiple eye drops tended to peak at night and that it was impossible to estimate the peak IOP based only on an IOP measured during daytime because there was no relationship between daytime and nighttime IOP [20]. In the present study, we measured 24-hour IOP variation in patients with POAG treated with triple eye drops and investigated the relationship with patient background factors including spherical equivalent.

## 2. Methods

Seventy-four outpatients (aged 54.6 ± 12.4 years, 37 males and 37 females) with POAG at the Department of Ophthalmology, Tokyo Metropolitan Police Hospital, Japan were studied. We randomly chose the right or left eye in the patients who had glaucoma in the both eyes. The subjects gave consent to be hospitalized for 24-hour IOP measurement. All patients were treated with three different types of eye drops: PG (latanoprost, travoprost, tafluprost, or bimatoprost), *β*-blocker (0.5% timolol maleate or 2% carteolol hydrochloride), and CAI (1% dorzolamide or 1% brinzolamide). The combinations of eye drops used by the patients are shown in Table 1.

The diagnostic criteria of POAG were as follows: normal open-angle; characteristic glaucomatous optic neuropathy with diffuse or focal optic rim thinning, cupping, or nerve fiber layer defects indicative of glaucoma and corresponding visual field changes according to Anderson and Patella criteria [21]; and presence of no other ocular, rhinological, neurological, or systemic disorders potentially causing optic nerve damage. Exclusion criteria were a history of cardiac or respiratory disorders; severe corneal disease, uveitis, or previous eye surgery; and concomitant use of any systemic medication that might affect the IOP.

We used the data of the patient that did not conflict with the above-mentioned exclusion criteria among the patients which have measured the IOP of 24-hour of treated with triple eye drops. Patients were hospitalized for 24-hour IOP measurement. In all patients, the IOP was measured in the sitting position by one ophthalmologist using a Goldmann applanation tonometer (Haag-Streit, Bern, Switzerland) averages of 3 times at the following hours: 10:00, 13:00, 16:00, 19:00, 22:00, 1:00, 3:00, and 7:00. Five glaucoma specialists were involved in this study to measure the IOP of 74 eyes. During hospitalization, patients self-administered eye drops. For nighttime IOP measurements, patients were waken gently and walked 10–20 meters to the tonometer. The patients returned to bed immediately after the IOP measurement.

**Figure 1:**
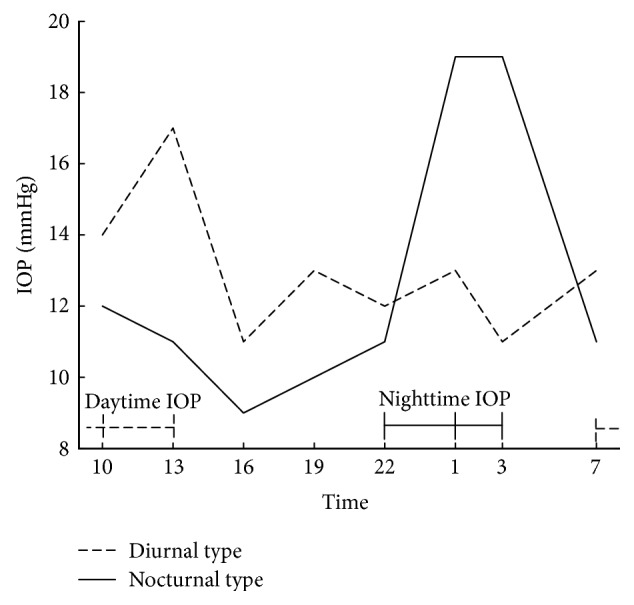
Typical examples of diurnal type and nocturnal type IOP profiles. Dashed line shows 24-hour IOP in a representative subject of diurnal type. In this patient, the averaged IOP for 7:00, 10:00, and 13:00 (daytime IOP) was higher than the averaged IOP for 22:00, 1:00, and 3:00 (nighttime IOP). Solid line shows 24-hour IOP of a representative subject of nocturnal type, in whom nighttime IOP was higher than daytime IOP.

To facilitate assessment of the relationship between 24-hour IOP variation and demographic and clinical characteristics (age, difference between peak and trough IOP, baseline IOP at 10:00, and spherical equivalent refraction), we classified the patients into two groups: diurnal and nocturnal types. In diurnal type, daytime IOP (averaged IOP for 7:00, 10:00, and 13:00) was higher than the nighttime IOP (averaged IOP for 22:00, 1:00, and 3:00). Whereas nocturnal type, the nighttime IOP was higher than daytime IOP (Figure 1).

A Humphrey Field Analyzer (Carl Zeiss Meditec, Dublin, CA, USA) was used with program 30-2 for evaluating visual field in the patients. The mean deviation averaged across all eyes was −11.3 dB. We measured objective spherical equivalent refraction with an auto refractometer (Nidek ARK-530-A®). For statistical analysis, the repeated measures ANOVA, the Mann-Whitney *U* test, and two-way repeated measures ANOVA were used at a significance level of *p* < 0.05 (two-sided test). Statistical analysis was conducted using SPSS (SAS Institute, Cary, NC) and Matlab® (The MathWorks Inc Natick, MA).

## 3. Results

**Figure 2:**
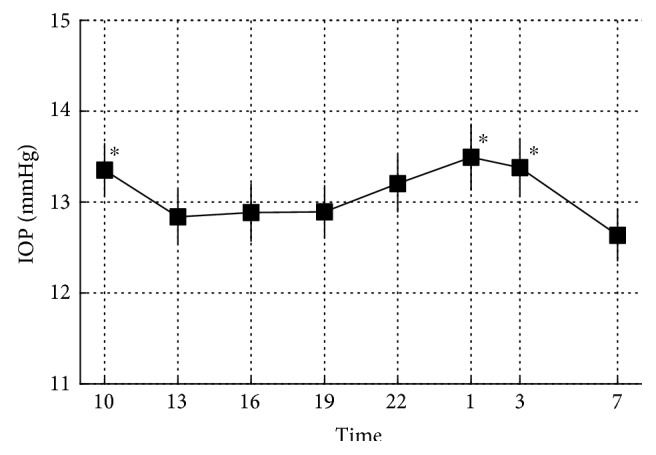
Mean 24-hour IOP at all time points of measurement in 74 eyes of 74 patients. Square indicates mean IOP of all eyes. The IOP at 7:00 was significantly lower than that at 10:00, 1:00, and 3:00. Error bar indicates standard deviation. ^∗^Higher than 7:00 (*p* < 0.05).

Figure 2 shows the variation of mean IOP at all time points of measurement for 74 eyes of 74 subjects treated with triple eye drops. The peak IOP was 13.5 ± 3.1 (mean ± SD) mmHg measured at 1:00, and the trough IOP was 12.6 ± 2.4 mmHg measured at 7 : 00. The trough IOP was significantly lower than the IOP at 1:00, 3:00, and 10:00 (*p* = 0.0066, 0.035, and 0.049, resp., repeated measures ANOVA).

**Figure 3:**
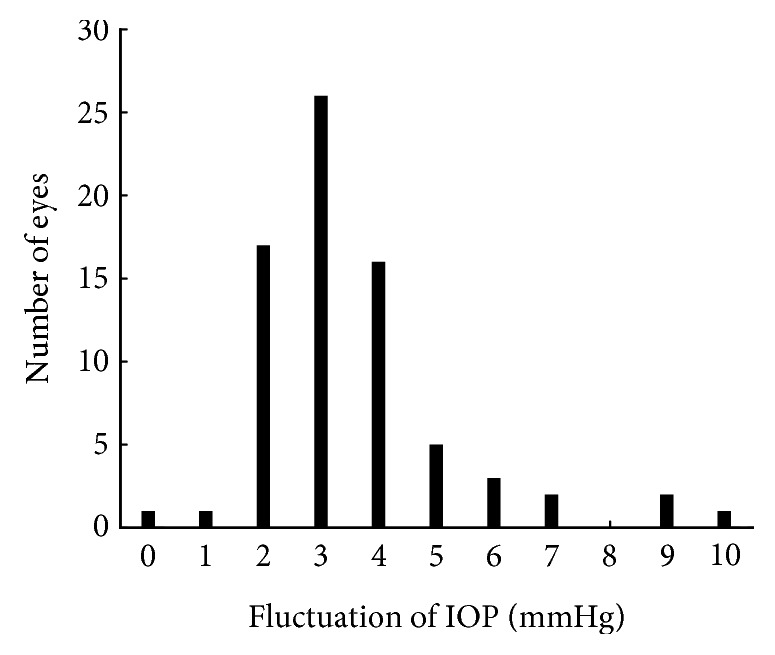
Distribution of 24-hour IOP variation among 74 eyes. Histogram shows the number of eyes with each variation.

Figure 3 shows the histograms of 24-hour IOP fluctuation defined as the difference between peak and trough IOP. The IOP fluctuation was within 10 mmHg in all subjects. Mean (± SD) 24-hour IOP variation was 3.3 ± 1.5 mmHg (95% confidence interval: 3.16–3.96 mmHg). The majority of 24-hour IOP fluctuation ranged from 2 to 6 mmHg. The 24-hour IOP fluctuation was 3 mmHg or more in 55 eyes even though all patients adhered to treatment with triple eye drops.

**Figure 4:**
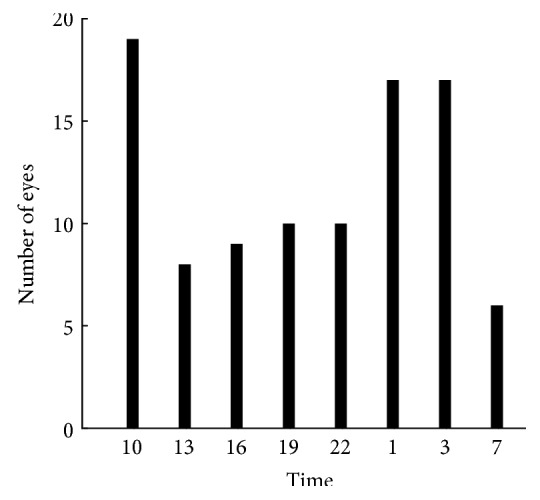
Time when peak IOP was measured. Histogram shows the number of eyes with each peak IOP time.

The histogram in Figure 4 shows the time when the peak IOP was measured in 55 eyes with IOP fluctuation of 3 mmHg or more allowing repetition in one eye that showed two or more peaks (Figure 4). The peak IOP was measured in 19 eyes at 10:00 (19.8%), 17 eyes at 1:00 (17.7%), and 17 eyes (17.7%) at 3:00. Sixty eyes (62.5%) did not have peak IOP during office hours. Note that the time of peak IOP was outside clinic hours in many eyes.

We compared diurnal type to nocturnal type to assess the relationship between 24-hour IOP variation and demographic and clinical characteristics. Eyes with 24-hour IOP fluctuation of 2 mmHg or less (*n* = 9) were excluded in subsequent analyses because the aim of the subanalysis is to assess causes of the 24-hour IOP variation in the patients. Eventually, 65 eyes were analyzed; 28 and 37 eyes belonged to diurnal type and nocturnal type, respectively (Table 2).

Based on the classification, the average IOP at 10:00 in diurnal type was significantly higher than that in nocturnal type. Moreover, the spherical equivalent and IOP variation in nocturnal type were significantly greater than those in diurnal type (*p* < 0.05). There were no significant differences in age and mean deviation of HFA between diurnal and nocturnal types.

To perform a subanalysis according to spherical equivalent, we classified into three groups: high myopic, moderate myopic, and low or nonmyopic groups. We compared the 24-hour IOP variation between the high myopic (HM: less than −6D, 26 eyes) and low or nonmyopic groups (LNM: more than −2D, 24 eyes).

**Figure 5:**
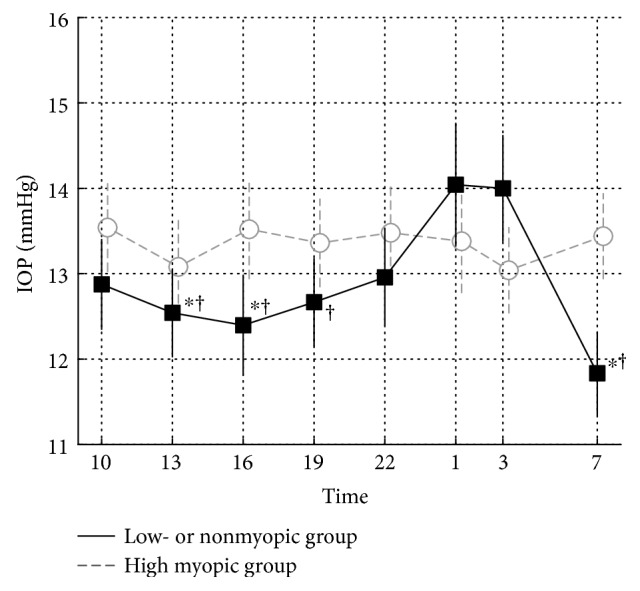
Comparison of 24-hour IOP variation in two groups with different spherical equivalent refraction. Gray dashed line and circles indicate mean IOP in high myopic group (HM). Black solid line and squares indicate mean IOP in low- or nonmyopic group (LNM). Error bar indicates standard deviation. ^∗^Lower than 3:00 (*p* < 0.05). ^†^Lower than 1:00 (*p* < 0.05).

Figure 5 shows the mean 24-hour IOP of HM and LNM. The 24-hour IOP variation in HM was relatively small. On the other hand, the 24-hour IOP in LNM tended to increase at nighttime. There were no significant differences between two groups at all time points. Only in LNM, however, mean IOP at 13:00, 16:00, 19:00, and 7:00 was significantly lower than that at 1:00 and/or 3:00 (*p* < 0.05, two-way repeated measures ANOVA). The IOP at 3:00 was significantly higher than the IOP at 13: 00, 16:00, and 07:00. Additionally, the LNM IOP at 3:00 was significantly higher than the IOP at 13:00, 16:00, 19:00, and 7:00, whereas there was no significant difference in HM group.

## 4. Discussion

This study determined the 24-hour IOP variation in patients treated with triple eye drops. The IOP measured in the sitting position normally peaks in the morning and declines toward the evening [10]. In patients on triple eye drop treatment, although the IOP level was higher at 10: 00. Compared with other time points during the day, the peak was at 1:00 (13.5 ± 3.1 mmHg) and IOP remained at a significantly higher level at 3:00 compared to other time points.

Similar to our study, Nakakura et al. examined the 24-hour IOP variation in patients on triple eye drop treatment and found that IOP was the highest outside clinic hours in 66.2% of patients, while IOP was the lowest during clinic hours in 72.5% [20]. The present study also found that the highest IOP was outside clinic hours in 62.5% of subjects, which was in good agreement with the findings of Nakakura et al. These results suggest that triple eye drop treatment markedly changes the pattern of the 24-hour IOP variation and reduces the IOP variation, but is associated with a risk of the IOP elevation during nighttime (outside clinic hours). Moreover, Konstas et al. reported that the IOP with multiple eye drops elevated at night, and the peak IOP was 14.2 ± 3.8 mmHg at 2:00 measuring the 24-hour IOP [22].

The mechanism by which IOP tended to increase at nighttime in patients treated with triple eye drops is not known. Since the parasympathetic nervous system becomes dominant during the night, the IOP-lowering effect of *β*-blocker may be reduced during the nighttime, resulting in a tendency of the IOP increase at night [16, 23]. However, Gulati et al. examined the effects of a PG, *β*-blocker, and CAI on the aqueous humor dynamics in patients with ocular hypertension and found that all agents had a smaller effect during the night than during daytime [24]. It is possible that individual IOP-lowering effects of PG, *β* blocker, and CAI are attenuated during nighttime, and concomitant use of these three agents may have further reduced the effect at night. Larsson et al. also reported that the effect of PG peaks 10–12 hours after administration [25]. Thus, the IOP-lowering effect of PG could be suboptimal during nighttime in patients who were administered PG eye drop at night.

In this study, subjects with high myopia (HM) and those with low or no myopia (LNM) were compared in a subanalysis. There were no significant differences in the IOP between the two groups at all time points, but the daytime IOP tended to be lower in the LNM group than in the HM group. In addition, a significant rise in the IOP was observed during the night in the LNM group. Previous literatures have suggested that IOP tends to be higher in myopic patients who have a long eye axis, than in emmetropic or hyperopic patients who have a short eye axis [26, 27]. We found similar results during daytime in patients on triple eye drop treatment, but the IOP at 1:00 and 3:00 was higher in the LNM group.

Loewen et al. studied three groups of healthy individuals with spherical equivalent ≥ +1D (hyperopia), −2D to 0 (emmetropia), and ≤−3D (myopia), comparing visual field during daytime in sitting position and during nighttime in supine position, as well as the 24-hour IOP in supine position [11]. They found more prominent variation in the hyperopia group than in the other groups, and nighttime increases in IOP in the former group. Similarly, we found a tendency toward nighttime IOP increase in patients with hyperopia, even though all subjects were on medications and IOP was measured in the sitting position.

The mechanisms underlying nighttime IOP increase in hyperopia individuals remain unclear, regardless of nontreatment and treatment. Read et al. reported that the eye axis length reaches a minimum during the night and that the eye axis length correlates with daily IOP variation [28]. Such circadian changes in the eye axis length may have induced nighttime IOP increases in hyperopia individuals in whom the eye axis length is already short. Although all patients were examined by gonioscopy and those with synechial angle closure were excluded from this study, it is possible that the nonmyopic subjects with a shallow anterior chamber were prone to have functional angle closure causing increases in outflow resistance and nighttime IOP.

There are some limitations to this study. This study examined 24-hour IOP variation in patients treated with triple eye drops, but subjects with progressive visual field loss despite therapy may have been included. Other limitations include variations in eye drop type and number of eye drop instillations among subjects. In this study, we measured IOP in the sitting position at all time points. Significant elevation of IOP in the supine position during sleep at night has been reported [29–31]. There is no doubt that measuring IOP in different measurement positions in daily life is important in studying 24-hour IOP variation. However, a Goldmann applanation tonometer is still considered to give a greater precision and has remained the clinical standard for the care of glaucoma patients, and Liu et al. reported IOP variation of the sitting position to predict the IOP variation in the supine position [32]. We think that it is meaningful to know the tendency of nighttime IOP by sitting position measurement with triple eye drop treatment. Further studies are needed to answer the following intriguing questions: how IOP changes depending on the measurement position in daily living and how nighttime IOP elevation impacts the progression of visual field loss.

## 5. Conclusions

We measured 24-hour IOP variation in POAG patients treated with triple eye drops (PG, *β*-blocker, and CAI). The peak IOP was observed outside clinic hours in many eyes. Furthermore, our study showed that nighttime IOP increases even during triple eye drop treatment, especially in patients with spherical equivalent of −2D or less. Because changes in IOP could be a potential risk factor for progression of visual field loss [33, 34], careful observation is required especially for low- or nonmyopic patients who are at risk of nighttime IOP elevation even during triple eye drop treatment.

## Figures and Tables

**Table 1:** Combinations of eye drops used by the patients in this study. Data is expressed as number of patients. All patients were treated with three different types of eye drops; PG, CAI, and *β*-blocker, in different combinations.

Prostaglandin	Latanoprost		Travoprost	Tafluprost	Bimatoprost
*β*-blocker	Carteolol hydrochloride 2% 2 times/1 time	Timolol maleate 0.5% 2 times/1 time	Carteolol hydrochloride 2% 1 time	Carteolol hydrochloride 2% 2 times/1 time	Carteolol hydrochloride 2% 2 times/1 time
CAI					
Dorzolamide 1%	4/1	5/10	1	1/0	1/0
Brinzolamide 1%	11/4	7/22	3	0/1	2/1

**Table 2:** Comparison of background factors between diurnal and nocturnal types of IOP profile. Data are expressed as mean ± SD. Significant differences in spherical equivalent, variation of IOP and IOP at 10:00 were observed between diurnal and nocturnal types.

	Diurnal type (*n* = 28)	Nocturnal type (*n* = 37)	*p* value
Age (y)	51.9 ± 10.5	56.1 ± 14.1	0.18
Spherical equivalent (D)	−5.7 ± 4.0	−4.0 ± 4.0	0.014
Mean deviation (dB)	−10.8 ± 8.6	−11.7 ± 9.3	0.81
24-hour fluctuations of IOP (mmHg)	3.1 ± 1.1	4.2 ± 2.0	0.01
IOP at 10:00 (mmHg)	14.1 ± 2.4	12.7 ± 2.4	0.01
